# Modelling Cell Polarization Driven by Synthetic Spatially Graded Rac Activation

**DOI:** 10.1371/journal.pcbi.1002366

**Published:** 2012-06-21

**Authors:** William R. Holmes, Benjamin Lin, Andre Levchenko, Leah Edelstein-Keshet

**Affiliations:** 1 Department of Mathematics, University of British Columbia, Vancouver, British Columbia, Canada; 2 Biomedical Engineering, Johns Hopkins University, Baltimore, Maryland, United States of America; Princeton University, United States of America

## Abstract

The small GTPase Rac is known to be an important regulator of cell polarization, cytoskeletal reorganization, and motility of mammalian cells. In recent microfluidic experiments, HeLa cells endowed with appropriate constructs were subjected to gradients of the small molecule rapamycin leading to synthetic membrane recruitment of a Rac activator and direct graded activation of membrane-associated Rac. Rac activation could thus be triggered independent of upstream signaling mechanisms otherwise responsible for transducing activating gradient signals. The response of the cells to such stimulation depended on exceeding a threshold of activated Rac. Here we develop a minimal reaction-diffusion model for the GTPase network alone and for GTPase-phosphoinositide crosstalk that is consistent with experimental observations for the polarization of the cells. The modeling suggests that mutual inhibition is a more likely mode of cell polarization than positive feedback of Rac onto its own activation. We use a new analytical tool, Local Perturbation Analysis, to approximate the partial differential equations by ordinary differential equations for local and global variables. This method helps to analyze the parameter space and behaviour of the proposed models. The models and experiments suggest that (1) spatially uniform stimulation serves to sensitize a cell to applied gradients. (2) Feedback between phosphoinositides and Rho GTPases sensitizes a cell. (3) Cell lengthening/flattening accompanying polarization can increase the sensitivity of a cell and stabilize an otherwise unstable polarization.

## Author Summary

Cell polarization is associated with intracellular gradients of signaling proteins such as Rho GTPases that organize the cytoskeleton in cell motility. We previously observed cells in microfluidic channels and studied their polarization and motility in a simplified (nearly 1 dimensional) geometry. There, precise gradients of chemically-inducible molecular probes were presented to elicit gradients of active Rac, independent of the upstream signaling. Here we develop a set of spatio-temporal mathematical models to account for the observed polarization behaviour of those cells, and their threshold response to induced Rac activity. These reaction-diffusion models for the interactions of signaling proteins (GTPases Rac, Rho, and Cdc42) and membrane lipids (phosphoinositides PIP, , ) are analyzed by a new method (‘Local Perturbation Analysis’) that explores the effect that pulses of stimuli have on local (global) variables, i.e. those intermediates that have slow (fast) rates of diffusion. Together, the models and experiments suggest that (1) spatially uniform stimulation makes the cells more sensitive to applied gradients. (2) Feedback between phosphoinositides and Rho GTPases sensitizes a cell. (3) Cell lengthening/flattening accompanying polarization can increase the sensitivity of a cell and stabilize an otherwise unstable polarization.

## Introduction

Many types of eukaryotic cells undergo directed motion in response to external spatial signals in a process known as chemotaxis. Before starting to move, a given cell polarizes according to directional cues in the environment, forming nascent “front” and “back” regions. At the front, actin cytoskeleton assembly powers protrusion, whereas at the back, actomyosin contracts and pulls up the rear. Orchestrating the localization of actin network regulators and myosin activators are signalling molecules such as Rho-GTPases and phosphoinositides (PIs). The spatio-temporal distribution of such regulatory molecules is thus critical to the correct polarization, motility, and chemotactic response of such cells.

Proteins of the family of Rho-GTPases (Rac, Rho, Cdc42) and the lipid PIs (PIP, 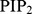, 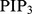), evolutionarily conserved across a wide range of eukaryotic cells, are implicated in cell polarization. These have garnered substantial interest as they are among the first elements in the chemotactic pathway to respond to a stimulus. Zones rich in Rac, Cdc42, 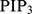 are associated with actin branching and growth, and zones rich in Rho are associated with myosin induced contraction. In many cell types, these zones are complementary, defining a “front” and “back” of the cell. Depending on cell type, the internal graded distribution of the GTPases and PIs amplifies shallow external gradients (of as little as 1–2% across the cell) into robust internal gradients [1]–[4]. The question of how such polarized distributions self-organize has attracted attention in both experimental and theoretical studies.

Motivating the theoretical development to be described in this paper, is a collection of microfluidic experiments outlined in [5]. In these experiments, mammalian (HeLa) cells were placed in narrow channels that constrain lateral movement and restricts them to a single dimension. The cells were modified so that diffusion-driven linear gradients [6] of a small molecule would induce translocation of the Rac activator Tiam1 to the plasma membrane; this resulted in graded Rac activation across the cell length independent of upstream effectors. Polarization and protrusion were observed in these experiments with variations depending on the slope and intercept of the applied stimulus and the strength of PI feedback.

Such experiments provide ideal testing ground for model development, refinement and validation. Our approach is to first consider the simplest hypotheses, reject those that are not supported by experiment, and successively build up the proposed network. Here we report in detail how models were constructed in a step-wise process to complement and crosscheck against these experimental observations. As the experiments also probed the effect of PI feedback on polarization, we are able to show agreement between theory and experiment linking GTPase and PI dynamics. To our knowledge, this is one of the first examples of such a match between GTPase-PI model predictions and observations.

Numerous models of GTPases and PIs have been proposed, but few have been developed in tandem with experiments. (See [7] for a recent review of qualitative models.) Models of the PI pathway are provided in [8], [9]. A model of Cdc42 in yeast cells is given by [10], [11]. Models of polarization via three interacting Rho GTPases include [12]–[14]. Some of these are based on a Turing mechanism [15] for spontaneous pattern formation. It was shown by Mori et al. [16], [17] in a reduced model with a single GTPase that rapid polarization can be achieved by “wave-pinning” as in [13], [14]. In this phenomenon, bistability drives the formation of a wave of activity that stalls due to substrate depletion. Models of this type are attractive since they can capture both sub-threshold (bistable) dynamics observed in [5] and noise sensitive (Turing) dynamics. Dawes et al. [18] connected the GTPase model [13] with a model of PI kinetics and explored the role of PI feedback. Marée et al. [19] have refined and studied this in depth in a 2D model of a motile cell. Numerous other models such as [8], [20], [21] consider fundamental aspects of polarization without identifying specific regulatory proteins. Some, such as [9] proposed a local excitation global inhibition (LEGI) model for the dynamics of PI3K, PTEN, and 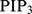 and found experimental agreement in amoeboid cells.

**Figure 1:**
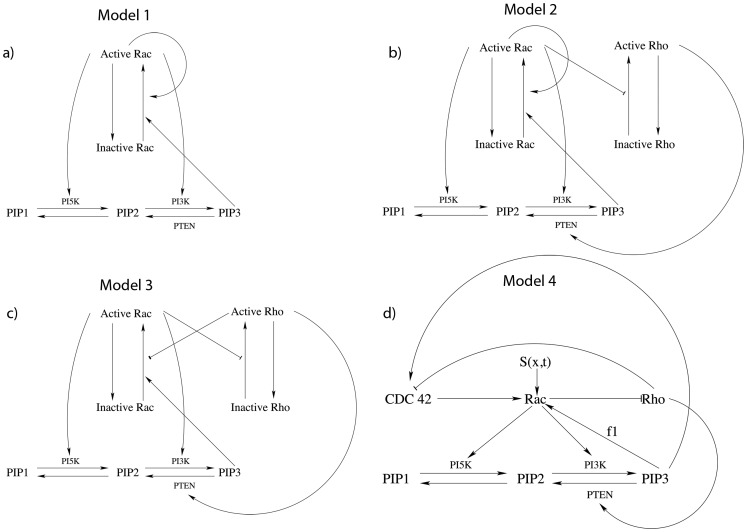
Schematics of a sequence of models explored in this paper. a) A basic single GTPase (“wave pinning”) module with crosstalk to the phosphoinositides (PIs). The GTPase module can only polarize on its own [16], but not when connected to PIs in this way. b) As before but with an additional passive Rho module: still no polarization possible with PI crosstalk. c) Mutual inhibitory Rac-Rho module: Polarization observed both with and without the PI layer. d) A more complete Cdc42-Rac-Rho module that exhibits polarization both with and without PIs. Model equations are shown in (1), (8), (16) and 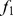 represents the strength of PI feedback to Rac. Arrows represent upregulation and bars represent inhibition. In all cases, proposed interactions between GTPases and PIs are taken from the literature [18], [24]–[28].

The availability of microfluidic cell polarity data provides a new opportunity to reconsider a variety of hypotheses in light of real cell behaviour. The essentially one dimensional geometry of the apparatus and direct activation of Rac, independent of upstream components, makes these particular experiments [5] amenable to model comparison. With this data, it is possible to revisit models that were purely theoretical so far, test their validity, and revise their structure. As explained below, this data quickly pointed to flaws in network connectivity that had been assumed in previous theoretical models, motivating the stepwise reconstruction of this connectivity. Here we develop a sequence of polarity models, starting with the simplest Rac-based polarization mechanism, and proceeding to include other GTPases that are widely known to be implicated in cell polarization and motility. For the simplest Rac-based model, Figure 1a, we rely on our previous theoretical work on “wave pinning” (WP) [16], [17]. This choice is motivated by observations [5] that cells display clearly distinct behaviours below versus above a threshold stimulus strength, a feature that the WP model recapitulates. Extending that work, we include interactions with phosphoinositides. In subsequent iterations, we incorporate the remaining GTPases Cdc42 and Rho. We focus on three particular experimental results: 1) the presence of a temporal bifurcation in motility response, 2) the apparent distinct functional effects of the input signal attributes (mean vs. gradient of Rac activation), and 3) the loss of response in some cases upon removal of PI feedback. We also explore the previously neglected effect of cell geometry, specifically cell aspect ratio, on polarization behaviour.

## Results

**Figure 2:**
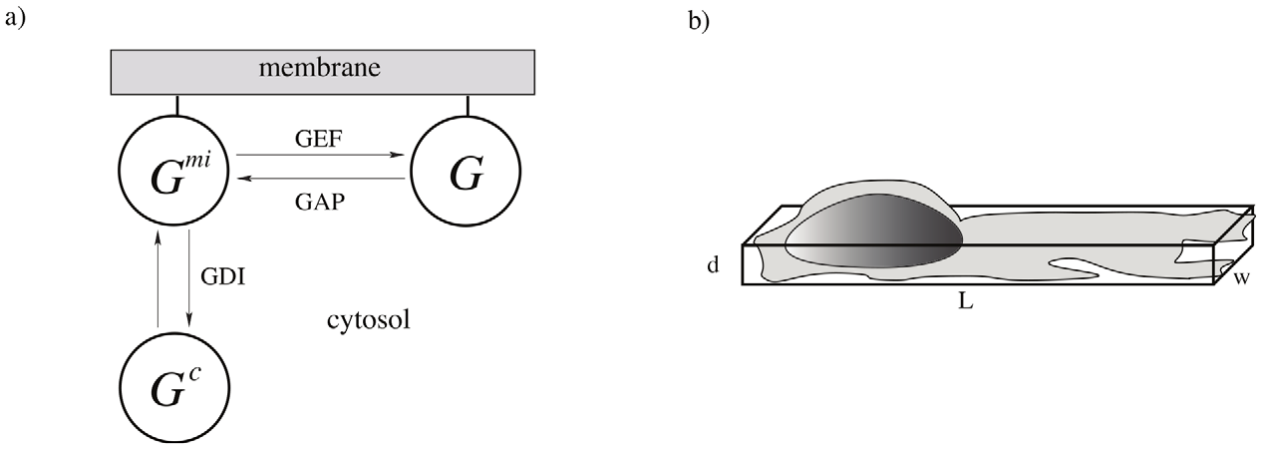
a) Membrane-cytosolic exchange for a single small GTPase. Activation and inactivation of membrane bound forms occur via GEF phosphorylation and GAP dephosphorylation respectively. The inactive form can cycle on and off the membrane aided by GDI's. b) Approximation of cell geometry with a box of dimensions 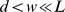. The width is constrained by the microfluidic channels in experiments [5].

The development of the model was guided by the experimental setup, and geared towards understanding the effects of the experimental manipulations, namely the role of signal parameters, phosphoinositide feedback, and length change observed in the responding cells. We consider the membrane-cytosolic cycling of the GTPases (Figure 2a), described in the next section. In view of the narrow channel confinement, we approximate cell shape by a box of length 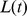, width 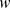, and depth 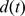 satisfying 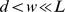 as shown in Figure 2b). The width is constrained by the channels, and assumed to be fixed. Cell elongation affects 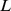 and 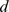 inversely since cell volume is roughly constant over the time frame of the experiments. The effect of depth “thinning” as a cell elongates proves to be significant to GTPase membrane cycling, as described below and in the Methods.

### Equations for membrane cycling of a single GTPase

Rho-GTPases are molecular switches that exist in both membrane-bound and cytosolic states. The membrane bound forms are activated by GEFs and inactivated by GAPs. Inactive GTPases are extracted from the membrane by GDIs and distribute in the cytosol (Figure 2a). In [5], endogenous Rac was activated by applying a gradient of rapamycin to HeLa cells that had two constructs. One of these was a fluorescently labelled Rac-GEF, and a second was a cell membrane anchor. Rapamycin acts to dimerize these two constructs and localize the GEF at the cell membrane where it can activate Rac. Our model will be formulated to take this Rac-GEF activation stimulus into account.

As the membrane-cytosol exchange of small GTPases plays an important role in the dynamics of these proteins, we first review aspects of the models that account for this cycling. This development follows [14], but emphasizes the effect of cell elongation that was not previously considered therein. We denote the concentration of a given GTPase by 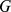 in its active membrane form and 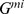, 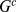 in the inactive membrane bound and cytosolic forms respectively. We make the biologically reasonable assumptions that each Rho protein has a constant total amount, 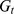, over the timescale of the experiments and that membrane cycling dynamics are much faster than activation/inactivation dynamics [22]. The latter hypothesis is a convenient simplification, that is not critical for model dynamics. As in [14], we write down a set of three balance equations for each GTPase, one PDE for each of the states defined above. (See the Methods for details, and Table 1 for meanings and values of all parameters.) Briefly, 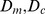 are membrane and cytosolic rates of diffusion of the GTPase, 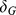 is GAP-mediated inactivation rate, 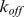 is the membrane dissociation rate, and 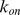 the membrane association rate. 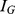 is a GEF-mediated activation rate that, we assume, depends on crosstalk. In each of the models we discuss, we provide the explicit assumption about the form of 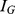 that captures the assumed crosstalk.

**Table 1:** Model parameters.

Parameter Name	Value	Meaning
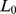	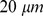	Baseline cell length
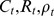	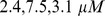	Total levels of Cdc42, Rac, and Rho
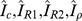	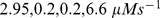	Cdc42, Rac, and Rho activation rates
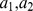	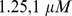	Cdc42 and Rho half max inhibition levels
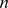	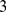	Hill coefficient for inhibitory connections
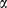	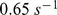	Cdc42 dependent Rac activation
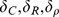	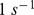	GAP decay rates of activated Rho-proteins
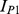	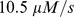	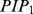 input rate
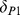	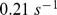	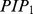 decay rate
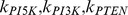	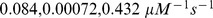	Baseline conversion rates
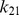	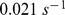	Baseline conversion rate
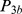	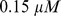	Typical level of 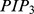
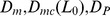	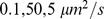	Diffusion Rates

Note that the parameters 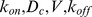 relating to membrane cycling of the three GTPases are not included due to their undetermined nature. The primary role of these parameters is to determine the quasi steady state fraction of membrane attached GTPases, which we instead account for by varying the composite parameter 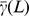.

In view of the small thickness of the cell, we neglect gradients in the depth direction and integrate in both depth (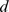) and width (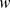) directions to arrive at a 1D spatial model. Cell length is retained as a parameter as discussed in the Methods. Adopting a quasi steady state (QSS) assumption that cycling between membrane and cytosol is very fast, we arrive at a model where each GTPase is assumed to have two forms, active (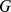) and composite inactive (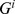). The latter is a sum of the inactive forms 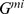 and 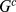 (projected from a 3D cell volume into the 1D spatial domain of the model, described in more detail in Methods). The GEF mediated reaction rates and “effective rates of diffusion” are modulated by cell geometry/length in the equations so obtained:
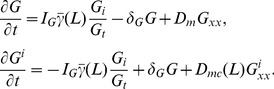
(1)where
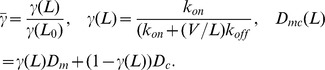
The parameter 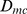 is a composite that weights the respective rates of diffusion of inactive GTPase forms by the average time spent on the membrane versus the cytosol. In [14], it was assumed that the GEF activation reaction could access the entire composite inactive pool 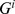. In reality, this reaction can only access the membrane bound proportion 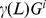. The incorporation of this feature into the model equations (8) will have a dramatic effect as discussed in ‘Hysteresis and the role of cell length’. Derivation of these model equations is found in Methods.

### Modeling the interacting GTPases, PIs, crosstalk, and feedback

While Turing instability is often invoked to account for spontaneous polarization [12], [20], this mechanism is not well suited for describing polarization of HeLa cells [5] or fibroblasts [23], which have a stable rest state and are polarizable by a sufficiently strong graded stimulus, but not by weak signals of small amplitude noise. In contrast, mechanisms based on Turing instabilities are sensitive to noise of arbitrarily small amplitude. We will refer to such cells having that property as ‘hypersensitive’. As HeLa cells are not hypersensitive, we here investigate only models where a threshold must be breached for a symmetry breaking event to occur. Mathematically, this threshold type response results from bistability.

In a spatial setting, models with bistable kinetics and diffusion can spawn waves of activity that initiate polarization. Typically, waves propagate into the domain from one or several initial foci. Halting the wave is essential to lead to a polarized domain, and this requires that the wave slows down and stops. This has been shown [16] to occur in conservative systems exhibiting a form of bistability, referred to as “wave pinning”. In this setting, a threshold based response initiates a wave and conservation leads to the depletion of an inactive substrate, stalling the wave and leaving regions of high and low activity separated by a narrow interface. This is the mechanism for polarization underlying the sequence of models discussed below. In addition to GTPases, PIs are known to play an integral role in symmetry breaking that was investigated experimentally in [5]. Here we describe the sequence of model explorations that led to the model adopted for the GTPase-PI signalling layers. We briefly describe the attributes of each model variant, but only the final version of the model is analyzed in full detail.

Phosphoinositides are membrane lipids that play well-known regulatory roles for the actin cytoskeleton. Both 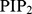 and 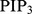 become highly expressed at the nascent front of a polarizing cell, and they interact with small GTPases and with actin-associated proteins. PIs are successively phosphorylated by kinases such as PI5K, PI3K and dephosphorylated by phosphatases such as PTEN (bottom layer of panels in Figure 1). The reaction-diffusion equations for the PIs are similar to those in [18], [19], and given in detail in the Methods. Proposed interactions between GTPases and PIs for all models are drawn from literature [18], [24]–[28]. The functions 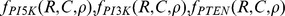 represent rates of phosphorylation by PI5K, and PI3K and dephosphorylation by PTEN and are assumed to depend on crosstalk from GTPases, as shown schematically in Figure 1. In testing the suitability of models described below, we studied properties both with and without feedback to/from the PIs.

### Preexisting GTPase-PI models (Model 0)

While there are many hypotheses for the crosstalk and interactions between GTPases and PIs, the actual network at play in any given cell type, subject to various stimulus types and conditions is generally unknown. We first considered a theoretical model proposed by Dawes et al. [18] (not shown) and its modification, studied in detail by Marée et al. [19]. This pre-existing model couples Cdc42-Rac-Rho GTPase dynamics with PI exchange and bidirectional feedback. Mutual inhibitory feedback between Cdc42 and Rho is assumed, as well as positive feedback from Cdc42 to Rac and from Rac to Rho. This was a reasonable first candidate for a model of HeLa cell polarization and motility. In both [18], [19], stimulus was assumed to flow via Cdc42 activation to other parts of the signaling pathways. However, the experiments reported by [5] shortcut the natural signal flow by directly activating a Rac GEF.

Incorporating this simple change in these previous models led to a surprising prediction that cells so stimulated should polarize in the wrong direction (opposite to the stimulus gradient). Thus, experimental data allowed us to reject this candidate pre-existing model. In hindsight, the reason for this is clear. In the original models, traversing the circuit from Rac to Rho to Cdc42 back to Rac encounters only a single negative feedback. Thus a mild stimulus-induced asymmetry in the Rac profile feeds back negatively onto itself. As this feedback is the source of amplification, it overpowers the original signal and leads to polarization in a direction opposing the initial stimulus. In view of the observation that HeLa cells polarize in the correct direction (up the gradient of Rac activator), we discarded these previous full models and decided to reconstruct a new model from the ground up. We use evidence from [5] and the broader literature on polarization as a basis to support or discard each model.

In the following sequence of models, we first asked whether a single GTPase coupled to PIs (Model 1) could account for major features of the data. We find that a single GTPase module can account for threshold based polarization with and without PI feedback. If PI feedback is added, the polarization capability is enhanced. However it is known that Cdc42 and Rho also participate in polarization. We next discuss Model 2 where Rho is passively coupled to the single GTPase polarization model (Model 1). For reasons discussed previously, we reject Models 1,2 as incomplete. In Model 3, a modification of an existing model in [13], Rac and Rho are assumed to inhibit each other. This model has the desired polarization properties, but it omits Cdc42, widely believed to be one of the master regulators of cell polarity/motility [29], [30]. Model 4, which is the subject of the remainder of the paper, maintains the structure of Model 3 while incorporating Cdc42 based on extensive background cell biology literature.

### Cooperative Rac (Model 1) with passive Rho (Model 2)

To assemble a new model, we started with the most basic relevant single-GTPase model due to [16], to which we added the appropriate feedback. We here identify the single GTPase with Rac, the target of chemotactic stimuli in the experiments of interest. The model for a single GTPase is a well studied cooperative feedback model whose mathematical workings (“wave pinning”) were described in [16], [17]. Adopting the same assumptions, we take Eqs. (1) with 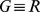 representing Rac, and Rac feedback onto its own GEF-induced activation as
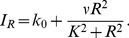
(2)(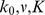 are constants representing basal activation, feedback-induced activation, and level of Rac for a half-saturated feedback activation via GEF.) In this model, a slow active form and fast inactive form interconvert. The active form feeds back onto its own production through cooperative binding. Inactivation is a first-order process. As discussed in [16], this system exhibits threshold behaviour, i.e. is consistent with a polarizable (rather than hypersensitive) cell in the appropriate parameter regime.

We connected the basic GTPase model to the model for PIs as in Figure 1a. We used Eqn. (16) with feedback terms
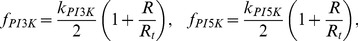
(3)and take 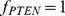. (Here 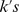 are phosphorylation rates, and 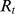 is total level of Rac in the cell.) We also assumed that PIs affect Rac dynamics by modifying (2) to
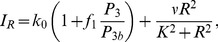
(4)where 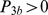 is some constant reference level of PIP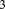 and 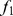 represents the strength of 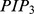 feedback to Rac activation. With parameters for the GTPase equations taken from [16] (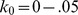, 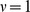, 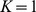, 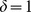, 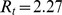) and PI-related parameters in Table 1, this model exhibits wave pinning based polarization for a range of feedback values 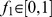 as required based on [5]. However, it is widely recognized that Cdc42 and Rho also participate in cell polarization, so we also consider a variety of possible connectivities that include these components along with Rac.

To avoid introducing too many features at once, we first consider a situation where Rac is a primary regulator that directs Cdc42 and Rho. Figure 1b illustrates Model 2, given by (1), (2), (6), (7), (16), where Rac directs Rho, both of which affect the PIs. To understand how this model behaves, first consider what happens in the absence of PIs. In that case, the Rac module is identical to Model 1 and the Rho module is “enslaved” to it. Rac polarizes and Rho sets up a complementary profile due to the negative feedback link. Now including PIs merely introduces a secondary positive feedback.

An important flaw in this model is that in the absence of PI feedback, Rho cannot influence Rac. While they are not specifically probed in the experiments that motivate these investigations, Rho and Cdc42 are observed to be more than passive regulators enslaved to Rac [29]–[31]. In this model, PI feedback between Rac, 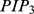, and Rho does form a complete circuit where Rho can influence Rac through 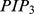. However it is observed in [5] that inhibition of PI3K, which reduces 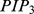 levels, does not destroy polarization. This suggests that a secondary feedback mediated by PIs is not the primary circuit linking the three GTPases. Thus, we do not consider Model 2 or any similar models where Rac unilaterally polarizes and directs the remaining GTPases as realistic. Additional experiments where Cdc42/Rac are experimentally inhibited or knocked out would provide a test of the hypothesis that they are members of a complete GTPase circuit as opposed to passive regulators driven by Rac. In the following iterations we consider minimal models that contain complete circuits.

### Rac-Rho mutual inhibition model (Model 3)

We adapted Model 2 by revising the number and types of feedback arrows to incorporate mutual Rac-Rho inhibition, as shown in Figure 1c. Without the PIs, this model recapitulates a first case studied in [13]. Here the model equations are (1) with 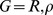 and
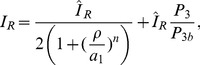
(5)

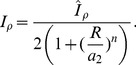
(6)(The constants 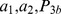 are typical values of Rho, Rac, 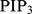 associated with a significant feedback on activation.) The second term in 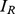 represents PI feedback to Rac. In this case, we use Eqs. (16), (3) and define
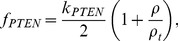
(7)to describe PI kinetics outlined in Figure 1c.

While this model appears to be schematically similar to Model 2, it incorporates an important structural difference. The bistability necessary for wave pinning to occur results from mutual inhibition (two negative feedbacks) as opposed to cooperative positive feedback. This is a natural next step in light of a result long posited by Thomas and recently proved [32] that bistability can result from networks with an even number of negative feedbacks while an odd number tends to yield limit cycles and other non-equilibrium dynamics. Reviewing Models 1–3, note that Model 1 had no negative feedbacks. The GTPase portion of the Model 2 effectively consisted of 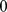 feedback loops as well. Since Rho was slaved to Rac the inhibitory link does not act as a feedback, and the circuit involving 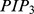 is a positive feedback loop. In model 3, the presence of 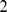 negative feedback loops led to the required bistable behaviour as discussed in [13].

Model 3 exhibits the following minimal required features to account for basic experimental observations on HeLa cells. (1) It has regimes with bistable kinetics needed for polarizability (as well as additional regimes of hypersensitivity in the Turing-instability sense). (2) It exhibits complementary localization of Rac and Rho, known to be related to protrusion and retraction respectively. This allows us to account for both “frontness” and “backness” cell attributes. (3) These behaviours occur both in presence and absence of PI feedback with all other system parameters held fixed, but can be “tuned” by the magnitude of that feedback.

### Rac-Rho-Cdc42 model with phosphoinositide feedback (Model 4)

In principle, Model 3 would comprise the minimal required model. For completeness, we added Cdc42, as shown in Figure 1d, given its importance as a master regulator [29]. However, results of Model 4 (described further on) also hold for Model 3.

We introduce Cdc42 with four criteria in mind. First, we sought interactions that lead to co-localization of Cdc42 and Rac that are complementary to the Rho profile. Second, we preserved the essential construction of two inhibitory connections of Model 3 to retain its bistable character. Third, we added a minimal number of overall GTPase interactions consistent with biological literature. Fourth, none of the GTPases is enslaved to the others. The model depicted in Figure 1d is the minimal possible model that satisfies these criteria. Removal of any connection or reversal of any feedback from positive to negative (or vice versa) destroys one or another of the required features, or requires additional compensating loops to avoid doing so. (Although a reversal of all three GTPase connections restores the required behaviour, it is contrary to biological literature showing positive feedback from Cdc42 to Rac.) We coupled the GTPase equations to the PI equations (16) with Eqs. (3), (7). The resulting model is described by (1) with 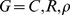 and
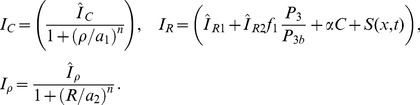
(8)The parameter 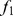 in (8) represents the strength of the feedback from 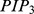 to Rac as shown in Figure 1d. The Rac-GEF parameters 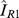, 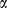, 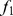, along with signal 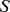 will be the target of further analysis with all other parameters left fixed. A more complete discussion of the forms of the GEF kinetic terms is given in [13] but it is important to note that 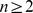 is required for bistability. Unless otherwise stated, this is the model we refer to from here on.

The GTPase part of this model consisting of Eqs. (1), (8) exhibits the bistability necessary for wave pinning to occur. To see this, consider the case of no PI feedback (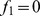) and no signal (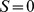) with 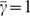, 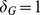. Set 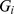 at its resting steady state value 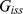 and define 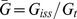. Now solve Eqs. (1), (8) for 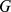 with 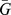 fixed as a parameter. Then it is straightforward to show that
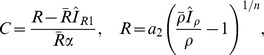
and
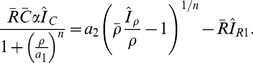
(9)


Define 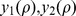 as the Rho-dependent expressions on the left and right hand sides of Eqn. (9), respectively. Then by plotting both together (with parameters in Table 1) in the 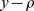 plane it can be shown that two stable steady states separated by an unstable repeller can exist for 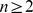. Furthermore, for suitable parameters, this can be made true for a range of values of 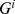. Thus the necessary conditions for wave pinning [16] are satisfied.

### Parameter values

The complete model contains numerous parameter values (Table 1). Many of their values are based on previous literature. We summarize the default values of basic rates and diffusion coefficients below, and then explain the procedure used to find interesting ranges of behaviour of the model when other key parameters were varied.

Consistent with [13], [14], [18], we take 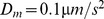, 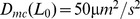. With assumed values of the necessary parameters, 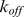 can be computed using
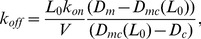
(10)completing the parameter set associated with membrane cycling. Given the undetermined nature of many of these parameters, we instead vary the composite parameter 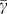 described in Methods, which represents the bulk effect of length variation in the model cell as it polarizes. GTPase crosstalk parameters are modifications of [14] to fit our system. PI parameters are a modification [18] by Marée et al. [19].

To gain insight into how parameter variations affect model behaviour, we utilized the ‘Local Perturbation Method’ described briefly in the Methods. This considers the stability of a homogeneous steady states against localized delta-function-like perturbations. The idea of the method is to replace the system of PDEs by approximating ordinary differential equations (ODEs) for local versus global variables (according to slow versus fast-diffusing intermediates). Then we can use bifurcation diagrams to explore the transitions between different regimes of behaviour. The LPA method allows us to detect both ultrasensitive and polarizable behaviour, a distinction of particular interest here.

**Figure 3:**
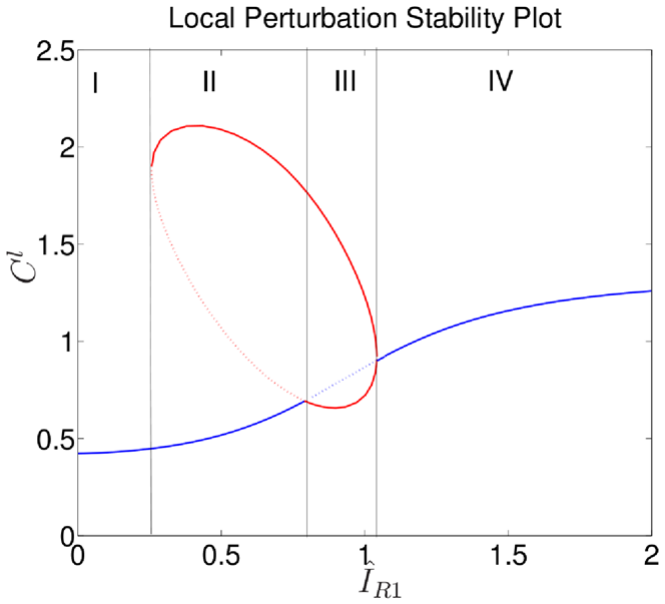
Basic “default” Local Perturbation Analysis (LPA) bifurcation diagram obtained using the LPA approximation of the PDEs (1), (8) using the reduction (18) (described in the Methods). Shown is steady state active (local) Cdc42 (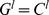) with 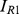, the basal Rac GEF activity level, as bifurcation parameter. Here 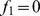 (no PI feedback), 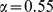 and all other parameters as in Table 1. The monotone increasing (blue) curve represents the HSS of the original system and is stable to homogeneous perturbations. Elliptical (red) arcs represent additional equilibria found in the LPA-system. Stability to small heterogeneous perturbations is indicated by solid lines vs instability shown by dotted lines. Region I is insensitive to perturbations, II is polarizable with sufficiently large perturbations, III is hypersensitive (Turing unstable), IV is insensitive but overstimulated. Similar results are seen when plotting 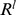 or 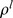 on the vertical axis.

With the above preparation, we now explore how specific aspects of the stimulus, the assumed feedback structure, and cell geometry affect the dynamics of the model 1D cell behaviour. Figure 3 maps out a typical parameter space structure for the discussed models. In the coming sections we discuss the relevance of each of these parameter regions and the bifurcations that occur between them.

### Stimulus magnitude and gradient

**Figure 4:**
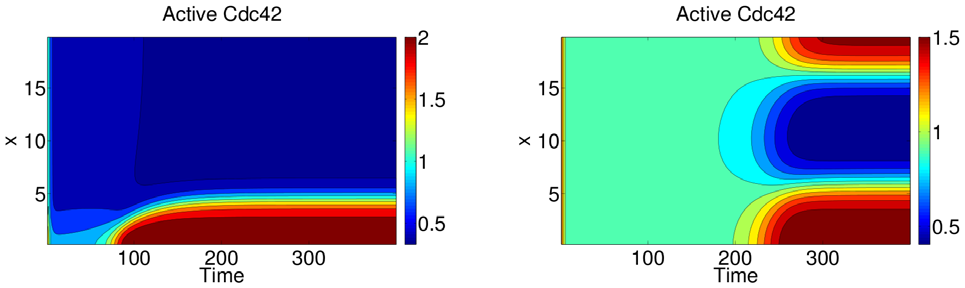
Kymographs (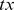-plots) of active Cdc42 concentration for the full PDE system with no PI feedback (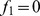), and parameters as in Figure 3. Left panel: 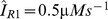 (Region II in Figure 3). Patterning is induced by a large local perturbation applied to active Rac at 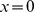. Identical behaviour is seen when this perturbation is applied to active Cdc42. Right panel: 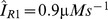 (Region III in Figure 3). Patterning is induced by random noise of size 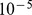 in the initial conditions. Similar (complementary) kymographs of Rac (Rho) are obtained (not shown).

Consider a cell, initially at rest, characterized by a low homogeneous steady state (HSS) of GTPase activity in Region II of Figure 3. Let the applied stimulus gradient be represented by 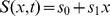. Recall that such gradients could be formed and maintained in experiments described in [5]. As in the experimental stimulus, we assume that this produces an internal Rac-GEF gradient. (A similar analysis can be performed with a Cdc42-GEF signal.) To polarize the cell, at least part of the cell domain must be elevated to Rac activity level above the threshold shown (dotted) in Region II of Figure 3. When this happens, that part of the cell evolves to a high Rac activity level (highest solid line, Region II), and, by virtue of diffusive coupling, creates a wave of activity that invades nearby portions of the cell. The wave stalls and leads to a polarized cell for parameter values in Region II (Figure 4, left).

Both the signal strength (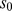) and gradient (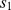) contribute to the ultimate response, but each plays a slightly distinct role. 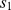 serves to produce an internal asymmetry in the GTPase profile and 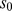 augments the size of the gap that has to be breached to induce polarization. In (8) the parameter 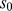 is directly added to 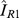, the bifurcation parameter in Figure 3. Thus, increasing 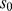 is equivalent to moving the state of the model cell to the right on that bifurcation diagram. This reduces the gap between the stable and unstable states and consequently the size of the perturbation required to induce polarization. Thus, 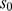 effectively controls the sensitivity of the cell to heterogeneous stimuli. 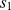, in contrast, produces the actual asymmetry necessary for the system to polarize. Numerical simulations of the full PDE system confirm this prediction of the reduced system. This sensitivity relationship and the functionally distinct roles of 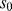 and 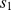 recapitulate the experimental observations in [5].

In the graded-stimulus experiments, a bifurcation occurred after some time. Stimulated cells had a long nascent period followed by an abrupt change to a much more active state. This suggests a temporal build up of Rac-GEF which sensitizes the cell. The resulting bifurcation would then lead to polarization. Other experiments and irreversibility of the stimulus-induced GEF activation [5] support this hypothesis.

### Exploring the feedback from Cdc42

**Figure 5:**
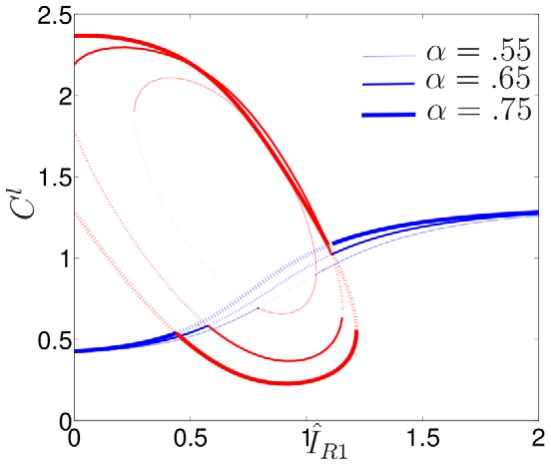
Effect of feedback from Cdc42 to Rac. LPA bifurcation diagram of (1) as in Figure 3, showing the effect of increasing 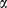 values. For larger 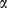 values, the model is more sensitive to heterogeneous stimuli.

We asked next how the positive feedback from Cdc42 to the Rac-GEF pathway affects model cell dynamics. This feedback is controlled by the parameter 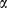. Figure 5 summarizes changes in the bifurcation structure of the reduced (LPA) model as this parameter is varied. We first decreased 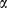 below 0.55 and noted that pattern forming capabilities of the system are completely lost.

Next, we increased this parameter. As expected, an increase in the strength of this positive feedback serves to sensitize the cell, i.e., increases the extent of the ultrasensitive Region III. For example, while for 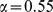 this region spans roughly 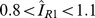 (bounded by intersections of thinnest monotonic curve with smallest ellipse in Figure 5), when 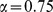, Region III has expanded to 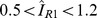. As 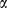 is further increased, Region II is squeezed into the negative 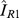 half plane, where it is no longer biologically feasible. Thus, Turing instability characterized by Region III takes over larger portions of the parameter plane. For 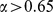 (for example when 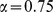 in Figure 5), a new regime forms between the original Regions III and IV of Figure 3. Here we find a new bistable region, with lower steady state (shown in red), higher one (blue) and unstable repeller (dotted red) in the approximate range 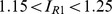. The size of this range grows in size as 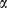 is increased. Unlike Region II of Figure 4 where a pulse of *activation* is needed to polarize, this new bistable region requires a pulse of *inactivation* (reducing the HSS below the dotted elliptical arc) to obtain polarization. (This prediction was verified with the full PDE system.)

### PI-feedback

**Figure 6:**
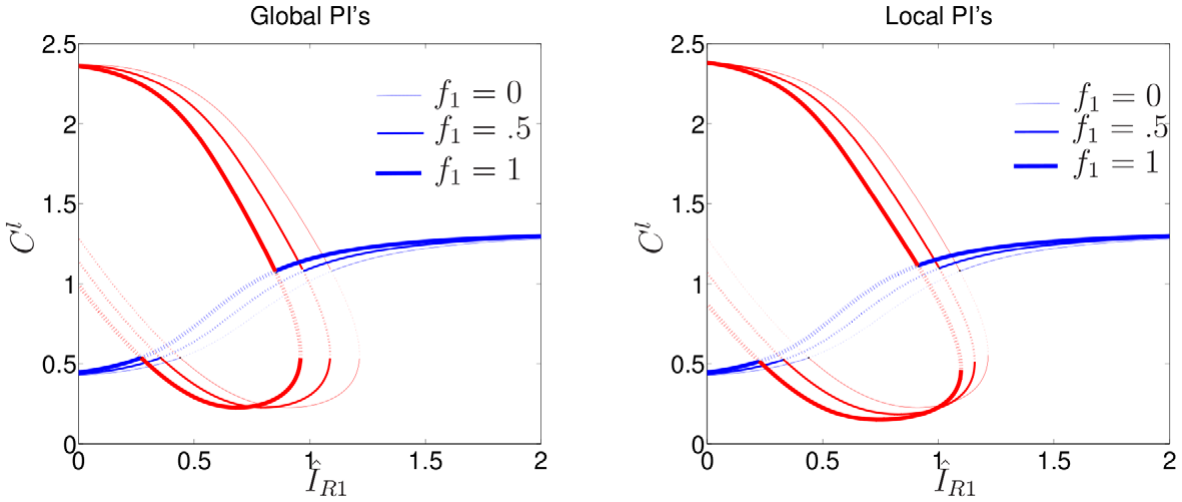
Effect of PI feedback to Rac. LPA bifurcation diagrams for (1) as in Figure 3, with 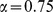 and multiple values of 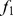. Left panel: PI variables treated as fast (global) LPA variables. Right panel: PI variables treated as slow (local) LPA variables. Note the simple linear leftwards shift as 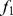 increases in both panels.

Experimental manipulations in [5] addressed the effect of a PI3K inhibition on the cells' response to graded stimuli. We used the full (9 PDE) model to address these observations. Having understood the behaviour of GTPase layer of signaling using the above analysis and simulations, we now turn to the full GTPase-PI feedback model. The parameter 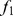 is used to tune the level of that feedback as shown in Figure 1. Recall that PIs are membrane-bound lipids. Their rates of diffusion are neither as fast as cytosolic GTPases, nor as slow as the membrane-bound GTPase forms. To gain some intuition using the LPA method, we therefore conducted two separate tests. We first treated the PI variables 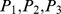 as fast (global) variables. The left panel of Figure 6 shows the effect of increasing the PI feedback parameter 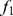 in this case. As seen, this produces a direct linear shift of the entire bifurcation plot to the left. This can be explained by the fact that in the infinitely fast diffusion limit for PIs, the feedback term 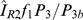 is spatially homogeneous, and therefore simply increments the bifurcation parameter 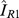. This can be interpreted as sensitizing the cell: for a given set of parameters, as 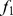 increases, the critical asymmetry required to produce polarization is reduced.

We next investigated the approximation that PIs are slow (local) variables, as shown in the right panel of Figure 6. While features of the two panels (global vs local) are not identical, qualitative aspects and, surprisingly, the dominant feature of leftward linear shift is preserved. This model prediction suggests that the primary role of PIs is to act as a global mechanism for increasing sensitivity.

**Figure 7:**
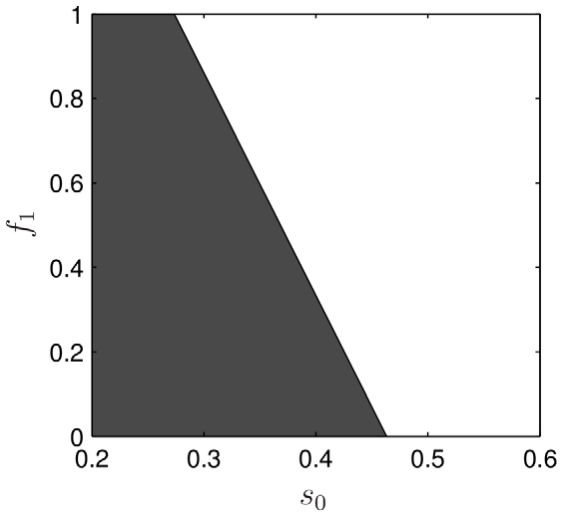
Two-parameter bifurcation plot for feedback from PIs to Rac (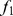) versus stimulus strength 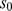 obtained via batch simulation of the full PDE system. 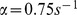
, and other parameters as in Table 1. The grey region is bistable and the white is Turing unstable. The linearity of this bifurcation curve is both qualitatively and quantitatively consistent with the linear shift of the bifurcation diagrams seen in Figure 6.

To check this prediction, we carried out simulations of the full 9 PDEs under systematic variation of the two parameters 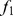 and 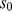. Results, shown in Figure 7 reveal a linear boundary separating bistable behaviour (“Region II”, shaded grey) from ultrasensitive behaviour (“Region III”, white). The linearity of this two-parameter bifurcation plot is consistent with the observed linear shift in Figures 6. Further, the total rate of shift in Figures 6 with respect to 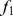 and the slope of the bifurcation line in Figure 7 are close to 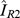, the parameter that controls the relative strength of this feedback. The combination of these three facts strongly suggests that the primary role of PI feedback is to provide global sensitivity. This feature is consistent with recent experiments in [5], and provides one of the strongest predictions of the model.

### Hysteresis and the role of cell length

**Figure 8:**
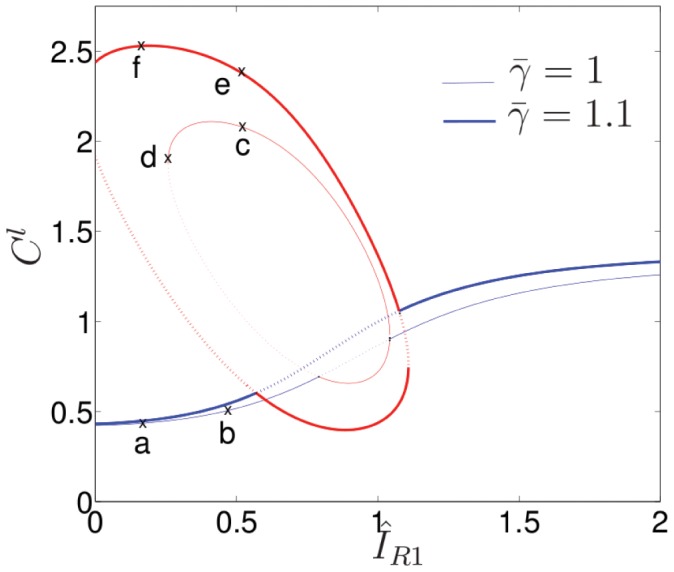
Effect of cell length: LPA bifurcation diagram with 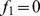, 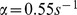, showing two values of 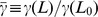. As 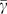 is increased, the stable Region I of Figure 3 at low 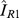 values vanishes, eliminating the hysteresis associated with the stable to bistable transition.

Experimental observations in [5] reveal that as a cell polarizes and elongates in the confined channels, its overall height changes inversely to its length. This feature was introduced into our models through volume conservation. Recall that the composite inactive form 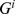 was introduced under a QSS assumption as a weighted sum of membrane bound and cytosolic inactive forms. These weights are explicitly linked to the geometry of the cell (details in the Methods) and can be explored consequently. As the model cell lengthens and flattens, the surface area to volume ratio increases. Given the form of 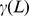 in our equations, this leads to a larger proportion of the inactive GTPase in the membrane bound form, resulting in two changes: (i) the composite form diffuses more slowly, and (ii) the GEF activation reaction can access a greater portion of inactive GTPase. However, whereas (i) has little effect, due to the relative insensitivity of the bistable and Turing unstable regimes to diffusion in the PDE system, (ii) has a substantial effect. As shown in Figure 8, increasing cell length tends to sensitize the model cell. This effect is similar to the effect of increasing either Cdc42 or PI feedback to the Rac-GEF pathway ( 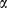, or 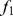). Meyers et al. [33] similarly considered the role of cell depth/length in polarization with a similar result that larger surface area to volume ratios lead to larger proportions of GTPases being in the phosphorylated active form. However they considered GEF's to be membrane bound and GAP's to be cytosolic where we consider both to be membrane bound. In either case, the end result is the sensitization of a cell as it flattens.

An additional feature seen here is the reduction and subsequent elimination of hysteresis as 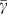 is increased. This hysteresis is present when the loop of steady states is entirely contained in the right half plane and a stable region for low values of 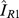 is present as with 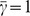. We refer to this as hysteresis for the following reason.

Consider, first, the following experiment with a resting cell of some fixed length. If 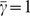, then the cell state is in the stable region (e.g., point 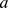 on Figure 8), where no heterogeneous signal can lead to polarization. Apply a signal of the form 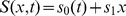 where 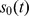 is an increasing function of time. This would be the case for a signal that cumulatively builds up over time. The buildup will cause the model to become increasingly sensitive to the applied asymmetry 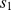 until it becomes sufficiently sensitive to respond/polarize. Graphically, the cell moves to point 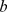 and subsequently 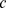 of Figure 8 upon polarization. Once polarized, the asymmetric component of the signal can be turned off (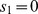) and the cell will stay polarized. As the background signal is washed out (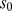 reduced and the state shifts leftwards on Figure 8), polarization will be maintained until the cell falls off the 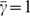 ellipse at point 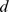 and again takes on a stable HSS at point 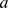. The state trajectory would follow the path 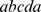 on Figure 8.

Interestingly, in addition to sensitizing the model, increasing length 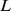 also removes hysteresis by pushing part of the loop into the left half plane and removing the stable region. A similar feature is seen in Figure 5 for 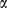 (and for other parameters we explored, not shown). However geometry/length is inherently a dynamic quantity whereas other parameters could be considered static on the time scales considered. So while genetic diversity in a host of parameters could play a role in the variability of behaviours among a population of cells or across cell lines, this length dependent removal of hysteresis can temporally stabilize an otherwise unstable polarization in a single cell.

Now consider the same experiment but with a cell capable of length change. Begin with a stable cell in a resting state (point 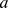) with the same cumulative stimulus 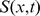. Again, after some point the cell will polarize (moving through point 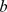 to point 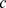 as before). As discussed previously, a static cell will lose polarization upon removal of this stimulus. In a dynamic cell however, such length change effectively shrinks or eliminates the stable region and associated hysteresis. When the cell lengthens, its state moves from 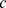 to 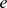 and, upon the removal of the stimulus, to 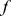. Thus, if the onset of polarization causes cell lengthening, the geometric effect described here affects internal signaling to stabilize the polarization, as indicated by the path 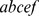 on Figure 8.

## Discussion

While the ultimate model we considered is a modification, extension, and rederivation of previously published models, it brings several new ideas and new results: first, all previous papers were theoretical, whereas here we were able to reassess details of the models in direct comparison with experimental data. Second, while previous models could account for polarization via Cdc42 stimuli, they produced incorrect predictions - thus invalidated - in view of that data, mandating a revision of the previously proposed GTPase connectivity. Third, using the novel LPA analysis, we have shown how the parametrization and analysis of behaviour could be accomplished with a novel analytic tool. Fourth, we provided here a hypothesis for how environmental factors can influence the response threshold through the GEF pathway. Fifth, and finally, we showed how the ratio of surface area to volume of the cell can influence the signalling.

We found that Model 4 is capable of qualitatively capturing many aspects of symmetry breaking and polarization in HeLa cells observed in microfluidic gradient generation experiments. We have included only features necessary to describe such observations. To aid the process of model development, model analysis, and parametrization, a novel analytic approximation technique, Local Perturbation Analysis, was introduced and applied. This proved to be fruitful as the model helped interpret experimental results and provided non-trivial insights into the behaviour of the experimental system.

The experiments were designed so as to allow convenient simplifications in modelling. The geometry of channels makes a 1D spatial representation both relevant and accurate. The tightly controlled gradient stimulus makes the assumption of (linear) signal shape appropriate. Finally, the stimulus bypasses a number of upstream signalling components and directly targets the Rac-GEF, making the input to the model clear and direct. Through these simplifications, we have produced a model that is both a reasonable representation of the system, and numerically and analytically tractable. This allowed for qualitative comparisons between model and experiment.

Because of the unique form of stimulation (via Rac, not Cdc42 activation), we could not directly use previously developed GTPase-PI model that had been tuned to stimulus inputs via Cdc42 GEFs. Rather than tinkering with that model we developed the new version from the ground up, proceeding from the simplest bistable GTPase module. A sequence of models involving one, two, or three GTPases with and without PI feedback were developed, allowing us to identify models with the minimal required capabilities. We showed that although the simplest model (with a single GTPase coupled to PIs driving polarization through positive feedback) does reproduce polarization (via “wave-pinning”) it is less suitable than models based on mutual inhibition since it does not incorporate the remaining GTPases, Cdc42 and Rho in the polarization process. In terms of complexity, the final variant (Model 4) consisting of three GTPases is a minimal mutual-inhibition model that mimics the typically observed GTPase localization behaviour, and accounts for the observed response to PI feedback tuning.

We investigated the roles of stimulus mean and gradient, feedback, as well as cell geometry using Model 4. Both full simulations of the model PDEs as well as bifurcation analysis of the LPA reduction provided insights. We found that signal mean could affect overall cell sensitivity while signal gradient drives the asymmetries needed to overcome a threshold for polarization. Further temporal buildup of Rac-GEF that results from a prolonged exposure to stimulus can account for bifurcations observed experimentally. This leads to the idea that that cells become increasingly sensitive with sustained stimulus, and is consistent with experiments. As far as the role of feedback between PIs and GTPases, we found that removal or reduction of PI feedback reduces sensitivity of the model cell to applied stimulus gradients. This, along with matching experimental results, supports the idea of feedback between PIs and GTPases (as opposed to PIs acting upstream of GTPases). Finally, we also found a role for changing cell geometry. When the cell lengthens, an increase in its surface area to volume ratio can remove hysteresis. This suggests that such purely geometric effects could stabilize otherwise unstable polarizations.

Limitations of the model include the absence of the cytoskeletal network, and possible feedback to and from that layer. In [19], we have shown that dynamic cell shape in 2D (top-down view of the cell) can feed back onto the internal biochemistry. Probing the multiple feedbacks and interactions in a similar 2D computational platform could provide new insights. In order to extend this work to other settings, it is important to similarly probe the Cdc42-GEF and/or Rho-GEF pathways (both experimentally and with a similar model) to more fully understand feedback to and from other GTPases. While the model was developed in the context of a specific cell type, many of its characteristics are observed in other cell lines. The model reductions and LPA approximation are also applicable to other settings. As more data regarding these types of signalling networks becomes available, these approaches will speed model development and aid in understanding the structure and dynamics of such networks.

## Methods

### Experiments

Experiments were performed by methods described in [5]. Briefly, constructs were introduced into HeLa cells, (a cytoplasmic YFP labeled TIAM1, a Rac GEF, conjugated to FKBP (YF-TIAM1) and Lyn11-FRB (LDR) that acts as membrane anchor) to directly activate Rac independent of upstream effectors [34]. HeLa cells were introduced into microfluidic chambers and allowed to settle (3–4 h). Linear gradients of rapamycin were created and maintained by actuation of flow in the microfluidic system. (The rapamycin dimerizes the constructs and leads to membrane-associated Rac activation.) Cells were imaged and observed over several hours, and classified according to initial and final polarization states. The PI3K inhibitor LY294002 was used to determine the effect of reducing feedback from PIPs to the GTPases.

### Model development and software

Models were formulated to describe the dynamic behaviour of these cells in several stages, as described in the main text. Detailed equations are provided in the following sections. Bifurcation diagrams were produced using MatCont [35], a numerical continuation package designed in MatLab (MathWorks). The full set of partial differential equations (PDEs) for each model were simulated using an implicit-diffusion explicit-reaction scheme with 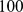 grid values. Example PDE simulations are seen in Figure 4.

### Derivation and reduction of the GTPase equations

In general, we consider up to three GTPases: Cdc42, Rac, and Rho, each of which is assumed to have three forms. Rather than writing all 9 PDEs, we here provide the form for a given GTPase, using the notation 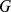 to represent any one of Cdc42, Rac, and Rho. Let 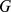 (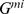) denote the level of active (inactive) membrane bound GTPase and let 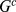 denote its cytosolic form. The total amount of GTPase in all these forms, 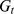, is assumed to be constant over the domain on the timescale of the experiments. Based on the schematic shown in Figure 2a), we write the set of equations
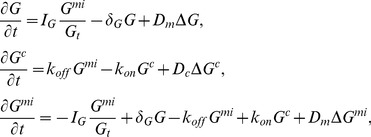
(11)where 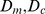 are membrane and cyosolic rates of diffusion, 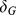 is GAP-mediated inactivation rate, 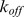 is the membrane dissociation rate, and 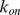 the membrane association rate. 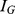 is a GEF-mediated activation rate and depends on crosstalk assumed in the specific models discussed.

Based on the 1D experimental geometry and controlled stimulus, it is reasonable to neglect gradients in all but the length direction. Define a 1D projection of the variable 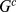 as
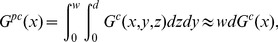
(12)where 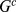 is approximated as nearly uniform across the width and depth directions. Physically, 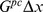 represents the number of molecules in a slice of width 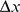 within the cell. It follows that
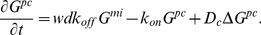
(13)As 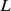 (but not 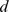) is directly observable experimentally, we rewrite 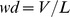 to eliminate the less readily measurable cell depth.

We now invoke the assumption that cycling between membrane and cytosol is very fast to make a quasi steady state (QSS) assumption. Then the fractions of the inactive form on the membrane and in the cytosol are, respectively, 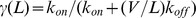 and 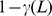. We now define a composite inactive form 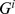 by
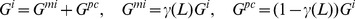
(14)and an “effective diffusion constant”
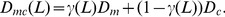
(15)The parameter 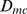 is a composite that weights the respective rates of diffusion of 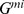 and 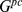 by the average time spent on the membrane versus the cytosol. With this reduction, we reduce the system of three equations (11) to a system of two equations in one space dimension and obtain Eqs (1). The normalization factor 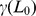 has been introduced to simplify parameter identification. We henceforth use the notation 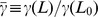.

### Phosphoinositide equations

Let 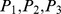 represent the phosphoinositides PIP, 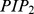 and 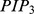. The interconversions of these are shown in bottom layer of each panel in Figure 1. We incorporate the feedbacks to phosphorylation by PI5K, and PI3K, and dephosphorylation by PTEN in the functions 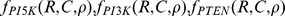. The set of equations adopted for the PIs are similar to those in [18], [19],
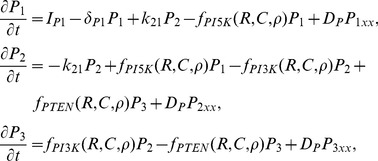
(16)

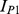
 is a constant source of 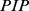, and 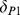 a constant rate of decay. All PIs are assigned the same rate of diffusion, 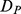.

### Local perturbation analysis (LPA)

We briefly outline the LPA method first introduced in [36]. The method simplifies the system of PDEs by considering the limit of infinitely fast diffusion of inactive GTPases 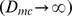 and infinitely slow diffusion 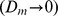 of the active GTPases. Under this limit, the full system of PDEs can be reduced to a system of ODEs that provide information about the initial growth of perturbations. This diffusion limit is particularly relevant to small GTPases where rates of diffusion of cytosolic and membrane bound forms vary by 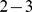 orders of magnitude.

**Figure 9:**
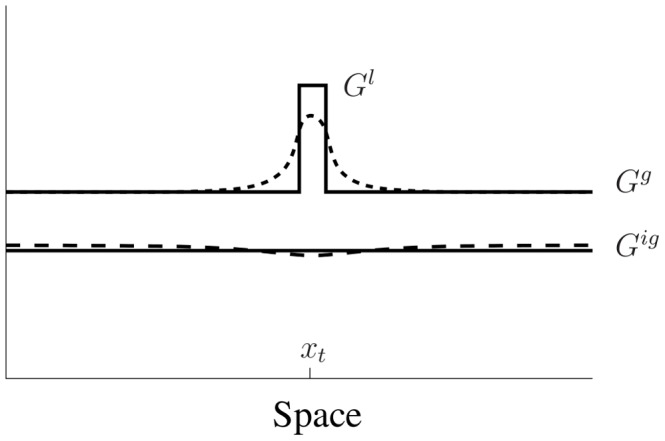
Schematic of the applied local perturbation in the LPA method. 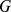
 represents the slow diffusing active form which has a local component 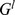 near the applied pertubation at 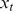 and a global behaviour 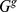 away from it. Since diffusion is slow, they do not directly influence each other on a short time scale. 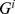 is fast diffusing and takes on only a global behaviour away 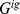. Solid curves qualitativly represent this pulse in the idealized diffusion limit and dashed curves represent the same situation with finite rates of diffusion.

Now consider a small perturbation that leads to localized high activation of the GTPase (square pulse in Figure 9). In the given diffusion limit, the active form 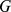 will take on a local behaviour near the pulse, and some uniform global behaviour far away. We denote those levels by, respectively, 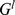 (local) and 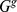 (global) as indicated in Figure 9. In the limit 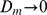 these two hardly interact. In contrast, in the 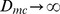 limit, the inactive form 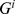 will take on a purely global behaviour 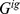, distributing the effect of the perturbation instantly. The PDE system (1) can then be approximated by the set of ODE's
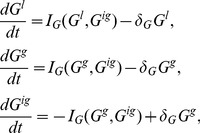
(17)for some initial time period until the perturbation is no longer localized. Applying the conservation of each GTPase and assuming the perturbation to be small in size yields 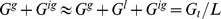. In this case 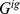 can be eliminated, leading to
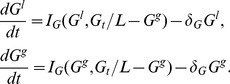
(18)A bifurcation analysis of the reduced ODE system provides clues as to how a localized perturbation will evolve over time in the PDE system. Even though the two mathematical structures are distinct, the large disparity in the true diffusion rates makes the LPA reduction a good approximation.

The bifurcation diagram in Figure 3 shows the results of this method applied to the GTPase system with no PI feedback (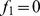). In this case the system of 6 PDE's reduces to 9 ODE's (3 for each GTPase) and, using conservation, further reduces to 6 ODE's (2 for each GTPase). The blue curve represents the steady states of the reduced system where 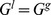. This is a solution of the well mixed system. It is also a homogeneous steady state (HSS) of the original PDEs that corresponds to a spatially uniform “rest state” of a cell before a stimulus (local pulse) is applied. Red curves represent additional states that can be reached by a highly localized patch while the bulk of the cell remains at its HSS. Dashed (solid) lines indicate that the state is unstable (stable) to arbitrarily small localized perturbations. While the details of the patterned states are not depicted in this type of bifurcation plot, the qualitative behaviour of the rest state and its response to a pulse can be seen.

Four distinct parameter regions are found: insensitive (I), polarizable (bistable) (II), ultrasensitive (Turing unstable) (III), and overstimulated (IV). Cells with states represented by Region (I) do not respond to a pulse stimulus, and return to the rest state rather than polarizing. Cells with state in Region (IV) have a uniformly high level of active GTPase throughout, and cannot polarize - they might typically flatten and protrude in all directions, but retain their uniform GTPase distribution. Region III represents cell states wherein polarization can occur spontaneously, or in response to noise of arbitrarily small magnitude. Finally, Region II represents cells that require a heterogeneous stimulus with a sufficiently asymetric profile in order to polarize. That is, the stimulus must be sufficient for part of the cell to breach some threshold(depicted by the dotted red elliptical arc).

Mathematically, these observations can be inferred from Figure 3 as follows. In the insensitive regions, there is a single HSS (single solid blue curve in Regions I, IV of Figure 3); this means that local perturbations or arbitrarily large amplitude decay back to that HSS and no spatial patterning can form. In the ultrasensitive region, the HSS (dotted blue line in Region III) is unstable to arbitrarily small heterogeneous perturbations, so that any noise will lead to new attractor states (represented by two solid red elliptical arcs in region III). In the polarizable region, the HSS is locally stable: both homogeneous and small heterogeneous perturbations decay back to this HSS. However, a sufficiently large local perturbation that increases the local level of one of the active GTPases beyond the threshold (dotted red elliptical arc in Region II, representing a repeller state) can induce patterning. The vertical distance between the HSS and repeller represents the magnitude of perturbation required to produce the spatially heterogeneous polarized state.

This analysis of the local-global LPA reduction provides insights, but is not fully predictive of the behaviour of the PDE system with finite rates of diffusion. The related collection of ODEs provides an approximation of the PDEs only as long as the perturbation is spatially localized. Once it spreads and a pattern begins to emerge, an asymptotic assumption that the integrated size of the perturbation be small fails and the approximation breaks down. Further, the bifurcation points present in the related ODEs are an approximation, rather than exact match, to full PDE bifurcation points. Thus, numerical simulations are necessary to provide a more complete understanding of the system.

Figure 4 shows numerical solutions of the PDE system in the 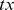 (“kymograph”) plane. Two pattern-forming regimes predicted in Figure 3 are ilustrated. In the bistable case (left), an initial local perturbation induces a wave that propagates into the domain and finally stalls, indicative of wave pinning. In the ultrasensitive regime, which is representative of noise sensitive cells, standard Turing patterning occurs where a wave with some dominant wave-number destabilizes the HSS and grows. Note that alternative techniques such as Turing stability analysis could be used to detect this regime. However, for our simplest model of 6 nonlinear PDEs, such analysis is challenging, and less revealing. LPA is a simpler alternative that provides an excellent numerical approximation for the Turing regime as well as its relationship to the WP regime. Finally, because our experimental cells have a stable rest state, the Turing regime is a less suitable regime to explore.
